# Bone scan index rise prior to osteonecrosis of the jaw with bone‐modifying agents in prostate cancer

**DOI:** 10.1002/bco2.70212

**Published:** 2026-04-28

**Authors:** Masaru Tani, Koji Hatano, Takashi Kamiya, Masatoshi Konishi, Atsuki Matsukawa, Hiromu Horitani, Syunsuke Inoguchi, Tomohiro Kanaki, Akihiro Yoshimura, Yuki Horibe, Yutong Liu, Nesrine Sassi, Toshiki Oka, Yohei Okuda, Gaku Yamamichi, Yu Ishizuya, Takuji Hayashi, Yoshiyuki Yamamoto, Taigo Kato, Tadashi Watabe, Atsunari Kawashima, Kayako Isohashi, Norio Nonomura

**Affiliations:** ^1^ Department of Urology The University of Osaka Graduate School of Medicine Osaka Japan; ^2^ Department of Medical Technology The University of Osaka Hospital Osaka Japan; ^3^ Department of Nuclear Medicine and Tracer Kinetics The University of Osaka Graduate School of Medicine Osaka Japan

**Keywords:** bone metastases, bone‐modifying agent, bone scan index, bone scintigraphy, denosumab, mandible, maxilla, medication‐related osteonecrosis of the jaw, prostate cancer, zoledronic acid

## Abstract

**Objective:**

This study aimed to evaluate the maximum bone scan index in the jaw (BSIJmax) before the development of clinical medication‐related osteonecrosis of the jaw (MRONJ) in patients with prostate cancer.

**Methods:**

We retrospectively analysed 135 patients with prostate cancer and bone metastases who received bone‐modifying agents (BMAs) between 2008 and 2025. Bone scintigraphy data were collected at baseline and during the BMA treatment. BSIJmax was calculated using computer‐assisted diagnostic software (BONENAVI). Primary endpoints were BSIJmax in the maxilla and mandible before the clinical diagnosis of MRONJ, compared with baseline values. Secondary endpoints included area under the receiver operating characteristic curves (AUCs) for diagnosing MRONJ using BSIJmax in the maxilla and mandible.

**Results:**

Among 135 patients, 27 developed MRONJ during a median follow‐up of 74 months (interquartile range, 41–124 months). Mandibular BSIJmax values before the clinical diagnosis of MRONJ were significantly higher than baseline values (0.090 vs. 0.027, *p* < 0.001), whereas maxillary values showed no significant change. The AUC for mandibular BSIJmax in diagnosing MRONJ was 0.803. A mandibular BSIJmax cut‐off of 0.033 yielded a sensitivity and specificity of 85% and 71%, respectively, for the diagnosis of MRONJ.

**Conclusion:**

The mandibular BSIJmax increased before the clinical diagnosis of MRONJ. The incorporation of BSIJmax into bone scintigraphy may enable early detection of MRONJ and timely intervention in patients with prostate cancer receiving BMA therapy.

## 1 INTRODUCTION

Approximately 50% of patients with prostate cancer and bone metastases experience skeletal‐related events (SREs) within 2 years, making preventing these events essential for preserving the quality of life.^1^ Bone‐modifying agents (BMAs), such as zoledronic acid and the receptor activator of nuclear kappa‐B ligand inhibitor denosumab, have demonstrated efficacy in reducing the incidence of SREs in patients with castration‐resistant prostate cancer (CRPC) and are widely recommended. Medication‐related osteonecrosis of the jaw (MRONJ) is one of the most serious adverse events associated with long‐term BMA therapy. Following its first report in 2003,^2^ the incidence of MRONJ has increased with the prolonged use of these agents.^3^, ^4^, ^5^ MRONJ becomes refractory to treatment after it develops; early detection remains the most effective strategy to prevent disease progression.^6^ The American Association of Oral and Maxillofacial Surgeons (AAOMS) revised the MRONJ staging system in 2009 by adding stage 0 to the existing Stages 1–3 and subsequently introduced risk categories in 2014,^7^, ^8^ recognising the importance of earlier diagnosis. However, the current system relies primarily on clinical findings, which may delay diagnosis despite these refinements.^8^, ^9^

Bone scintigraphy is a well‐established imaging modality for detecting bone metastases and assessing skeletal disease activity. Furthermore, it offers greater sensitivity in detecting subtle metabolic, vascular and pathophysiological bone changes compared with anatomical imaging techniques, such as radiography, CT or MRI.^10^, ^11^, ^12^ Several studies have demonstrated the utility of bone scintigraphy in diagnosing MRONJ.^13^, ^14^ Furthermore, the bone scan index (BSI), a quantitative parameter derived from computer‐aided diagnostic (CAD) software, has been validated for assessing skeletal tumour burden in metastatic prostate cancer.^15^, ^16^

Recently, applying this concept to the jawbone has been greatly considered, with a focus on the maximum BSI value in the jaw (BSIJmax) which has been proposed as a potential early biomarker of MRONJ.^6^, ^17^, ^18^ Bone scintigraphy is routinely used for follow‐up in patients with prostate cancer and bone metastases; however, studies specifically focusing on BSIJmax in this population are limited. Furthermore, its clinical utility in predicting MRONJ has not been established, and longitudinal changes in BSIJmax before clinical MRONJ remain unclear. In this study, we aimed to evaluate BSIJmax before the clinical diagnosis of MRONJ in 135 BMA‐treated patients with prostate cancer who underwent serial bone scintigraphy. In addition, we analysed the changes in BSIJmax from baseline to the prediagnosis of clinical MRONJ to assess its potential utility for the early detection of MRONJ.

## 2 PATIENTS AND METHODS

### 2.1 Study design and data collection

In this single‐centre, retrospective, observational study, we aimed to evaluate the utility of BSIJmax in predicting MRONJ in patients with castration‐sensitive prostate cancer (CSPC) or CRPC and bone metastases receiving BMA. A total of 197 patients were enrolled between January 2008 and April 2025. The inclusion criteria were bone scintigraphy performed within three months of prostate cancer diagnosis and during the BMA treatment period. Dental evaluation and care were coordinated with the primary dentist before initiating BMA therapy. Bone scintigraphy was performed every 3–6 months at the discretion of the attending physician. For analysis, bone scintigraphy conducted before the clinical diagnosis of MRONJ or at the last follow‐up was used. In addition, bone scintigraphy performed within 12 months before the clinical diagnosis of MRONJ was acceptable. Patients with jawbone metastases were excluded. In addition, data on concomitant or past systemic therapies, including first‐generation antiandrogens (bicalutamide and flutamide), second‐generation antiandrogens (abiraterone, apalutamide, enzalutamide), chemotherapy (docetaxel, cabazitaxel) and radium‐223, were collected from medical records. Data on invasive dental procedures during the study period were collected from medical records. In all patients who underwent invasive dental procedures, BMAs were temporarily discontinued around the time of the procedure in accordance with institutional practice. Ultimately, 135 patients met the eligibility criteria and were included in the final analysis (Figure S1). To evaluate longitudinal changes in BSIJmax, data from 37 cases (13 MRONJ and 24 non‐MRONJ cases) with available bone scintigraphy data at 5, 15 and 25 months before clinical MRONJ diagnosis or final evaluation were analysed. Bone scans for each time point were performed within a 5‐month window, before or after the target time point.

### 2.2 Bone scintigraphy

The patients received 555 MBq (15 mCi) of ^99^mTc‐methylene diphosphonate (FUJIFILM RI Pharma Co., Ltd., Tokyo, Japan) intravenously. Scintigraphy images were obtained 3‐h postinjection. Semiquantitative analysis of the jawbone tracer uptake was conducted using the BONENAVI software (Fujifilm Toyama Chemical Co., Ltd., Tokyo, Japan; EXINI Diagnostics AB, Lund, Sweden). The highest BSI value automatically generated for each jawbone region was defined as BSIJmax, calculated separately for the maxilla and mandible for analysis.

### 2.3 Endpoints

The primary endpoints were BSIJmax in the maxilla and mandible before the clinical diagnosis of MRONJ, compared with baseline values. The secondary endpoints included the areas under the receiver operating characteristic (ROC) curves (AUCs) for the diagnostic performance in identifying MRONJ using BSIJmax in the maxilla and mandible. In addition, longitudinal changes in BSIJmax were analysed in a subset of patients.

MRONJ was diagnosed by oral surgeons using the AAOMS classification system.^19^ Stage 0 was defined as no clinical evidence of necrotic bone but nonspecific clinical or radiographic findings. Stage 1 involved exposed necrotic bone or a fistula extending to the bone, without signs of infection. Stage 2 included an exposed necrotic bone or fistula with an infection, pain, erythema and possible purulent drainage. Stage 3 comprised necrotic bone with an infection and one or more of the following conditions: exposed necrotic bone extending beyond the alveolar bone, pathological fracture, extraoral fistula, oroantral or oronasal communication or osteolysis extending to the mandibular border or sinus floor.^19^

### 2.4 Statistical analysis

The BSIJmax values for the maxilla and mandible in the MRONJ group were compared at baseline and before clinical diagnosis of MRONJ. In the non‐MRONJ group, the BSIJmax values for the maxilla and mandible were compared at baseline and the most recent time point. Comparisons of BSIJmax values at baseline and before clinical diagnosis of MRONJ, or at the most recent time point, were performed using the Wilcoxon rank‐sum test. Fisher's exact test was applied for comparisons of categorical clinical characteristics between the groups. ROC analysis was performed to assess the predictive accuracy and calculate AUC values. All statistical analyses were performed with JMP software (Version 18.0.0; SAS Institute Inc., Cary, NC, USA). Statistical significance was set at *p* < 0.05.

### 2.5 Ethics

This study was approved by the University of Osaka Ethics Committee (approval number: 1339720) and conducted in accordance with the principles of the Declaration of Helsinki. We obtained written informed consent from all the study's participants.

## 3 RESULTS

### 3.1 Patient characteristics

**FIGURE 1:**
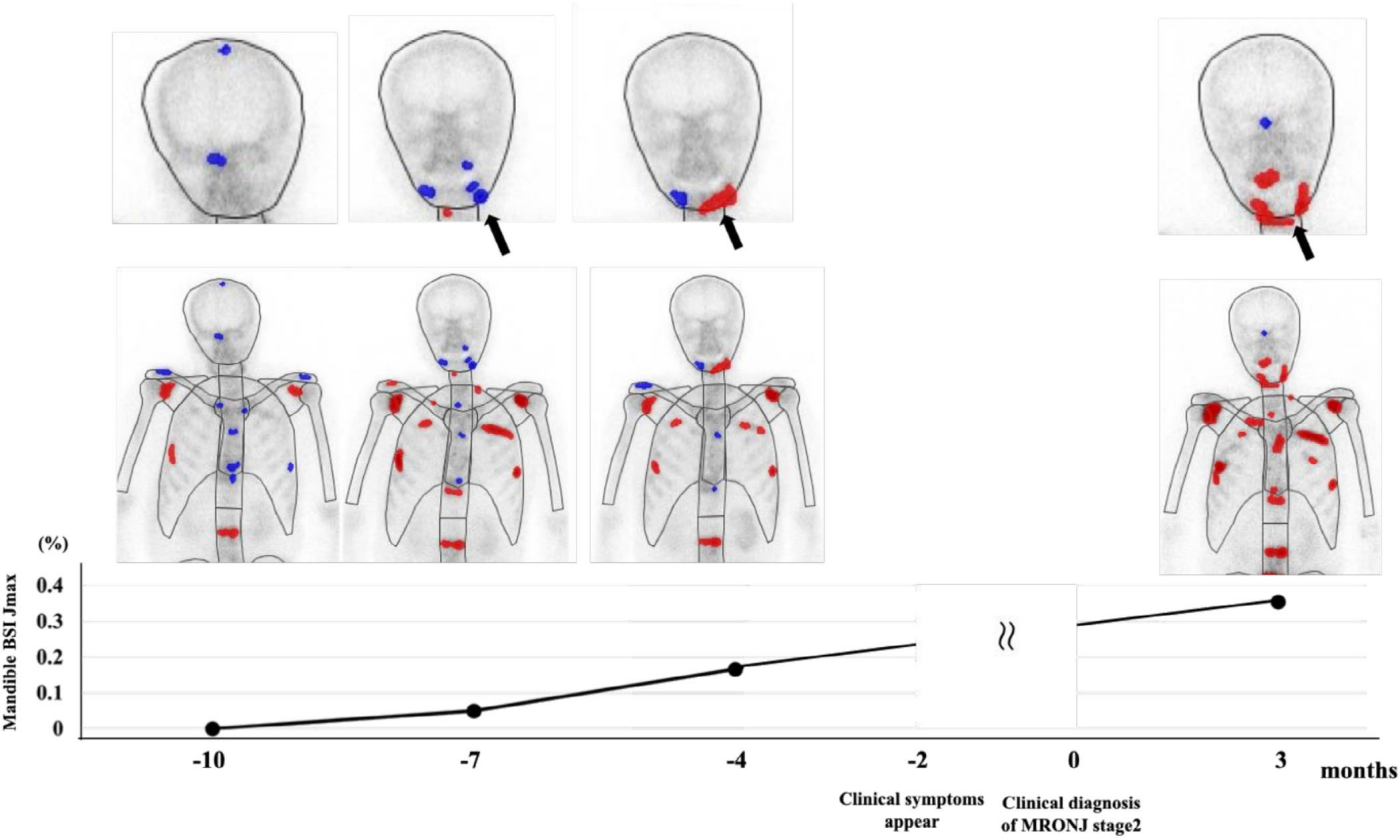
Representative case showing gradual increase in the maximum bone scan index in the jaw (BSIJmax) before the clinical diagnosis of medication‐related osteonecrosis of the jaw (MRONJ).

A total of 135 patients were included in this study, and their characteristics are summarised in Table 1. At the time of BMA initiation, 47 patients (35%) had CSPC and 88 patients (65%) had CRPC. We observed that 27 patients developed MRONJ, whereas 108 did not, during a median follow‐up of 74 months (interquartile range [IQR], 41–124 months). The median patient age was 71 years (IQR, 67–77 years). Zoledronic acid was administered to 79 patients (59%) and denosumab to 56 patients (41%). There were no significant differences between the MRONJ and non‐MRONJ groups in prior or concomitant systemic therapies for prostate cancer. Likewise, whether patients underwent invasive dental procedures did not differ between the groups. MRONJ occurred in the maxilla in six cases (22%), mandible in 20 cases (74%) and both jaws in one case (4%). MRONJ stage distribution was as follows: Stage 0, two patients (7%); Stage 1, five patients (19%); Stage 2, 13 patients (48%); and Stage 3, seven patients (26%). The median interval from the most recent bone scintigraphy to the clinical diagnosis of MRONJ was 4.9 months (IQR, 1.4–7.7). Increased tracer uptake in the jawbone was observed before the onset of the clinical MRONJ symptoms. A representative example is shown in Figure 1.

**TABLE 1:** Patient characteristics.

	Total *N* = 135	No MRONJ *N* = 108 (80%)	MRONJ *N* = 27 (20%)	*P*‐value
Median age, years (IQR)	71 (67–77)	72.5 (67.8–78)	70 (67–74)	0.079
Median BMI, kg/m^2^ (IQR)	22.2 (20.0–24.4)	22.0 (19.6–24.3)	22.6 (21.6–25.6)	0.099
Castration sensitivity, *N* (%)				0.240
CSPC	47 (35%)	35 (32%)	12 (44%)	
CRPC	88 (65%)	73 (68%)	15 (56%)	
BMA agent, *N* (%)				0.667
Zoledronic acid	79 (59%)	62 (57%)	17 (63%)	
Denosumab	56 (41%)	46 (43%)	10 (37%)	
Concomitant or past history of first‐generation antiandrogen, *N* (%)				0.301
Yes	126 (93%)	102 (94%)	24 (89%)	
No	9 (7%)	6 (6%)	3 (11%)	
Concomitant or past history of second‐generation antiandrogen, *N* (%)				0.240
Yes	88 (65%)	73 (68%)	15 (56%)	
No	47 (35%)	35 (32%)	12 (44%)	
Concomitant or past history of chemotherapeutic agent, *N* (%)				0.111
Yes	83 (61%)	70 (65%)	13 (48%)	
No	52 (39%)	38 (35%)	14 (52%)	
Concomitant or past history of Ra‐223, *N* (%)				0.894
Yes	16 (12%)	11 (12%)	3 (11%)	
No	119 (88%)	95 (88%)	24 (89%)	
History of invasive dental procedures, *N* (%)				0.231
Yes	16 (12%)	11 (10%)	5 (19%)	
No	119 (88%)	97 (90%)	22 (81%)	
Duration of BMA treatment		15 (8–29)	24 (17–70)	0.004
Location of MRONJ				
Maxilla			6 (22%)	
Mandible			20 (74%)	
Maxilla—mandible			1 (4%)	
MRONJ stage, *N* (%)				
0			2 (7%)	
1			5 (19%)	
2			13 (48%)	
3			7 (26%)	

Abbreviations: BMA, bone‐modifying agent; BMI, body mass index; CRPC, castration‐resistant prostate cancer; CSPC, castration‐sensitive prostate cancer; MRONJ, medication‐related osteonecrosis of the jaw.

### 3.2 BSIJmax before the clinical diagnosis of MRONJ

**FIGURE 2:**
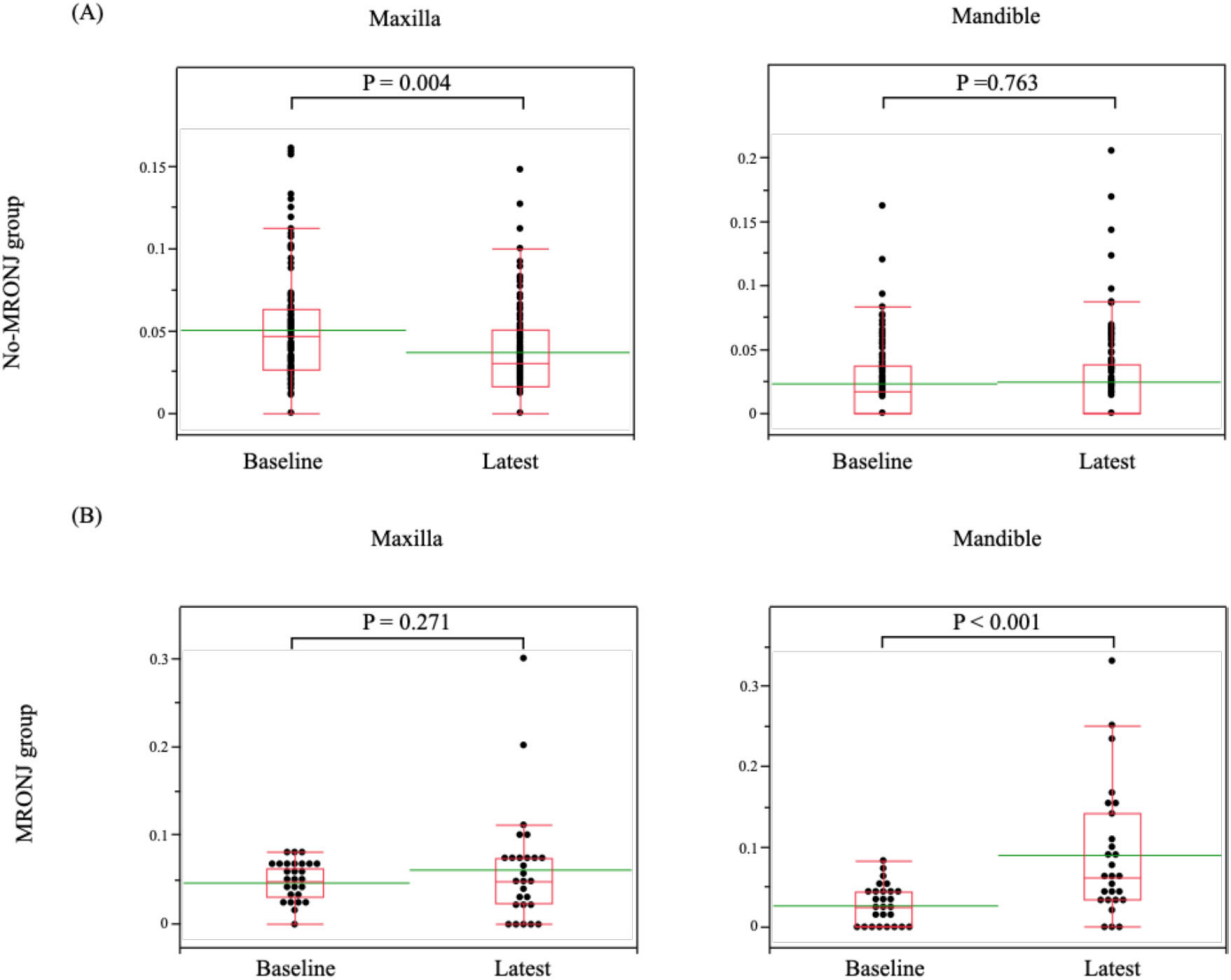
Comparison of baseline and latest maximum bone scan index in the jaw (BSIJmax) values. (A) Nonmedication‐related osteonecrosis of the jaw (MRONJ) group. (B) MRONJ group.

In the non‐MRONJ group, the mean BSIJmax did not increase at the last time point in the mandible from baseline before BMA administration (0.024 vs. 0.023, *p* = 0.763), and in the maxilla, it rather decreased (0.037 vs. 0.050, *p* = 0.004) (Figure 2A). In contrast, in the MRONJ group, the BSIJmax value in the mandible before clinical MRONJ significantly increased compared to baseline (0.090 vs. 0.027, *p* < 0.001), whereas the increase in the maxilla (0.061 vs. 0.047, *p* = 0.271) was not significant (Figure 2B). Subgroup analyses stratified by BMA types were performed. Mandibular BSIJmax was significantly higher in the MRONJ group than in the non‐MRONJ group in both the zoledronic acid and denosumab subgroups (0.088 vs. 0.029, *p* = 0.004; and 0.092 vs. 0.024, *p* = 0.044, respectively) (Figure S2A,B). No consistent or significant increase was observed in maxillary BSIJmax across BMA subgroups.

### 3.3 Predictive performance of BSIJmax for MRONJ

**FIGURE 3:**
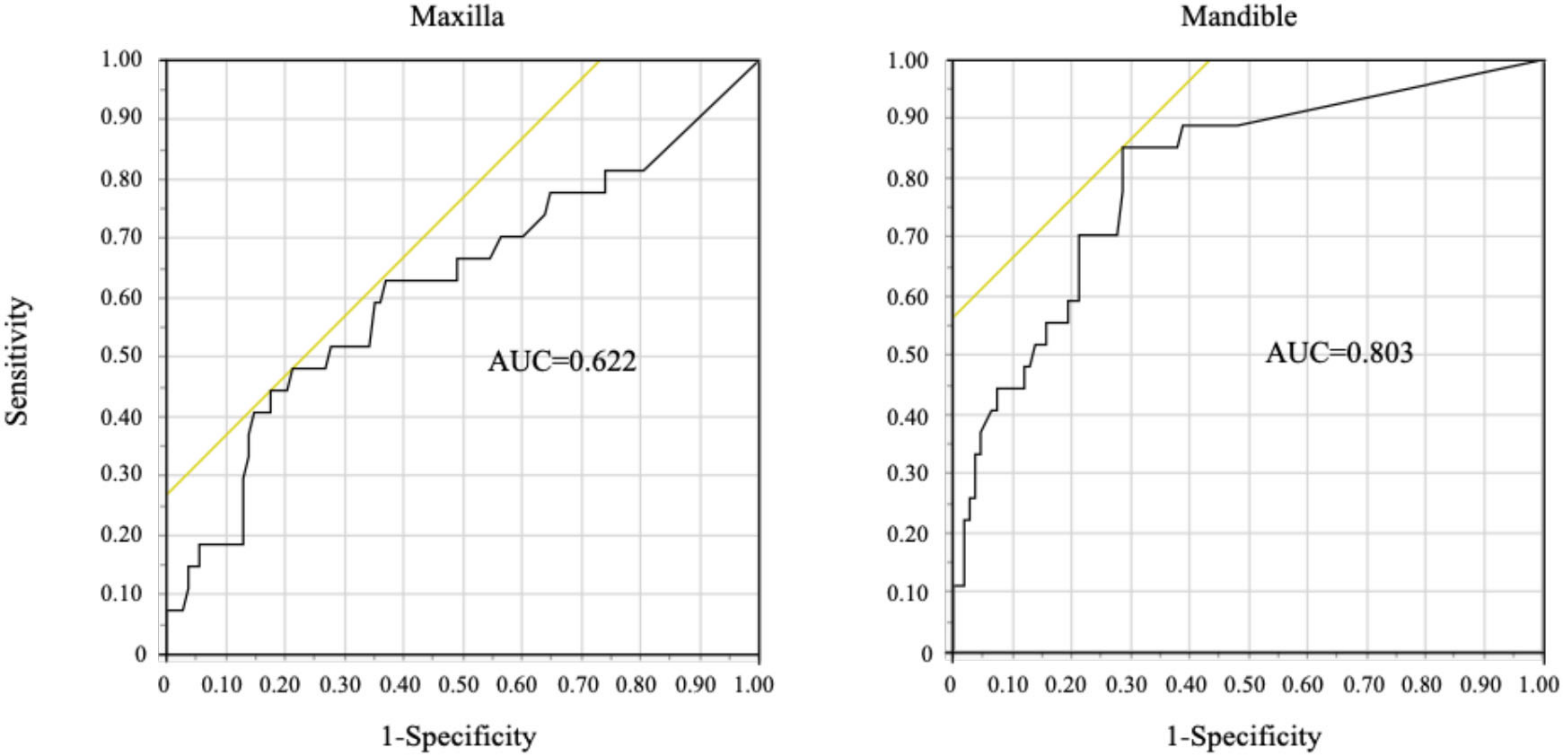
Receiver operating characteristic (ROC) analysis of the maximum bone scan index in the jaw (BSIJmax) for medication‐related osteonecrosis of the jaw (MRONJ)

ROC analysis was conducted to evaluate the predictive performance of BSIJmax for MRONJ (Figure 3). The AUC for BSIJmax was 0.622 and 0.803 for the maxilla and mandible, respectively.

A maxillary BSIJmax cut‐off of 0.054 yielded a sensitivity and specificity of 48% and 79%, respectively. The corresponding positive and negative predictive values were 36% and 86%, respectively. A mandibular BSIJmax cut‐off of 0.033 yielded a sensitivity of 85% and specificity of 71%, with corresponding positive and negative predictive values of 43% and 95%, respectively.

### 3.4 Temporal changes in BSIJmax

Longitudinal bone scintigraphy data from 13 patients in the MRONJ group and 24 patients in the non‐MRONJ group, with imaging available approximately 5, 15 and 25 months before clinical MRONJ diagnosis or final evaluation, were analysed to investigate the timing of BSIJmax elevation (Figure S3A).

At baseline, no significant differences were observed in BSIJmax between the MRONJ and non‐MRONJ groups in the maxilla (0.054 vs. 0.046, *p* = 0.316) and mandible (0.030 vs. 0.033, *p* = 0.730). Mandibular BSIJmax was significantly higher in the MRONJ group than in the non‐MRONJ group as early as 25–15 months before clinical diagnosis, with further elevation noted 5 months prior to diagnosis. In contrast, maxillary BSIJmax remained unchanged at all time points between 25 and 5 months before diagnosis.

When stratified by BMA types, the zoledronic acid subgroup demonstrated a temporal pattern comparable to that of the overall cohort, with earlier and progressive elevation of mandibular BSIJmax in patients who developed MRONJ (Figure S3B). A similar directional trend was observed in the denosumab subgroup; however, statistical significance was not reached, likely due to the smaller sample size (Figure S3C). No consistent temporal changes were observed in maxillary BSIJmax in either subgroup.

## 4 DISCUSSION

The findings of this study suggested that mandibular BSIJmax increased significantly several months before the clinical diagnosis in patients who developed MRONJ compared with those who did not. ROC analysis demonstrated good discriminatory performance of mandibular BSIJmax in identifying patients at risk of MRONJ. Longitudinal analysis further showed a progressive increase in mandibular BSIJmax over time in patients who developed MRONJ, indicating the presence of a subclinical disease preceding the onset of symptoms. These findings support that monitoring the BSIJmax dynamics may facilitate earlier identification of patients who could benefit from timely dental evaluation and preventive interventions.

MRONJ was first reported in 2003.^2^ The increasing incidence of any grade and severe MRONJ associated with long‐term use of BMA has become a growing concern.^3^ Treatment strategies for MRONJ are selected according to disease stage, with either conservative management or surgical intervention recommended accordingly.^19^, ^20^ Numerous studies have reported superior efficacy and prognosis with surgical treatment compared with conservative therapy, resulting in an increased preference for surgical approaches.^21^ Palla^22^ and Graziani^23^ demonstrated that surgical treatment in the early stage of MRONJ resulted in higher healing rates, lower invasiveness and better quality of life than surgery in the advanced stages.^24^ Moreover, advanced‐stage MRONJ is associated with lower healing rates even with conservative management.^24^ The healing rates after MRONJ onset are further reduced in patients with cancer compared with those without cancer, which may be due to factors such as systemic therapy, compromised general health, and high doses of BMA.^25^ These findings highlight the critical importance of early diagnosis and timely therapeutic intervention of MRONJ in patients with prostate cancer.

Conventional imaging modalities such as X‐ray, CT and MRI are useful for evaluating anatomical structures; however, they have a limited ability to detect early metabolic or vascular changes and are often susceptible to artefacts from dental prostheses. In contrast, bone scintigraphy offers higher sensitivity for detecting physiological alterations in the bone. However, image interpretation can be influenced by the variability in tracer dosage, timing of image acquisition and equipment.^17^ The introduction of CAD systems, such as BONENAVI, allows objective and reproducible quantification of tracer uptake through BSI, independent of the interpreter's experience.^26^ BONENAVI, widely used in Japan to evaluate bone metastases in prostate cancer, can be adapted to include BSIJmax analysis with minimal algorithmic adjustments, thereby facilitating clinical integration. From a technical perspective, BSIJmax can be extracted using the same CAD framework employed for whole‐body BSI calculation. In BONENAVI, jaw‐specific BSI values can be obtained without additional imaging acquisition, as the analysis is based on standard whole‐body bone scintigraphy data. Although region‐specific evaluation requires manual confirmation of the automatically segmented jaw region, no advanced programming or specialised expertise beyond routine BSI analysis is necessary. Importantly, similar quantitative approaches may be feasible using other BSI‐based software platforms. For example, VS BONE BSI (Nihon Medi‐Physics) also provides region‐of‐interest–based BSI analysis, suggesting that jaw‐specific assessment is not inherently limited to BONENAVI. Therefore, BSIJmax monitoring could be implemented in institutions equipped with commercially available BSI analysis software.

In this study, we confirmed that BSIJmax is effective for the early prediction of MRONJ in patients with prostate cancer who are routinely monitored using bone scintigraphy. The identified cut‐off values (0.054 for the maxilla and 0.033 for the mandible) were consistent with those reported in previous smaller‐scale studies.^6^, ^17^ Given the distinct mechanisms of zoledronic acid and denosumab, we performed BMA‐stratified analyses to assess potential heterogeneity. A preclinical increase in mandibular BSIJmax was consistently observed in both subgroups, suggesting that its elevation reflects a shared pathophysiologic process rather than a BMA‐specific effect. The concordant findings across BMA types support the robustness of mandibular BSIJmax as an early marker of MRONJ. Similar patterns were observed in the longitudinal subgroup analyses, with progressive mandibular BSIJmax elevation in the zoledronic acid subgroup and a comparable, though nonsignificant, trend in the denosumab subgroup. This consistency across BMA types reinforces the temporal relationship between mandibular BSIJmax elevation and MRONJ onset.

The latency period from pathological tracer uptake to the clinical diagnosis of MRONJ has been reported to average 14.4 months.^27^ The mean latency period in our study between the initial imaging detection and clinical diagnosis among the 27 patients who developed MRONJ was 4.9 months. Furthermore, evaluation of BSIJmax values at 15 and 25 months prior to diagnosis demonstrated that tracer accumulation in the jawbone occurred significantly earlier in the MRONJ group than in the non‐MRONJ group. These findings suggest that abnormal tracer uptake may precede clinical onset by a greater margin than that previously reported. The pathogenesis of MRONJ remains incompletely understood; however, it may involve several factors, including suppression of osteoclast activity and disruption of the bone remodelling cycle by BMAs, causing microfractures,^2^ as well as the anatomical susceptibility of the jawbone to infection due to its proximity to the oral microbiota.^28^ Bone scintigraphy can detect early inflammatory changes and alterations in local blood flow before the onset of clinical symptoms, indicating its potential as a useful tool for the early detection of disease progression to more severe stages.

In this study, the majority of MRONJ cases occurred in the mandible (74%) rather than the maxilla (22%), which is consistent with previous reports.^25^ Diagnostic performance of BSIJmax was higher in the mandible than in the maxilla, with greater sensitivity, specificity and predictive value, indicating that mandibular BSIJmax provides reliable diagnostic information for MRONJ. The superior performance in the mandible compared with the maxilla was likely due to the larger number of mandibular cases. These metrics were comparable to those reported by Pergolini et al.,^25^ further validating the robustness of these parameters for early MRONJ detection.

In addition, anatomical differences between the maxilla and mandible may influence baseline BSIJmax values. The maxilla is more vascularised with a thinner cortical bone compared to the mandible,^29^ which contributes to higher background tracer uptake.^6^, ^30^ Our study confirmed significantly higher BSIJmax values in the maxilla, even among patients who are MRONJ‐negative, consistent with previous reports, suggesting the need for site‐specific diagnostic thresholds. Notably, an increase in mandibular BSIJmax was observed in patients who developed maxillary MRONJ (data not shown), suggesting that elevated mandibular BSIJmax may also facilitate early prediction of MRONJ, including cases involving the maxilla.

Nonetheless, elevated BSIJmax may occur in common dental conditions such as periodontal disease or tooth extraction. Nonspecific tracer accumulation has been reported in 56%–72% of patients,^30^ many of whom had known risk factors for MRONJ. Therefore, increased jawbone uptake does not confirm MRONJ; nevertheless, it warrants careful clinical monitoring. From a practical perspective, an upward trend in mandibular BSIJmax during BMA therapy may serve as an early warning signal for impending MRONJ. Even in the absence of overt clinical symptoms, early referral for dental or oral and maxillofacial evaluation should be considered, together with reinforcement of oral hygiene measures and closer clinical surveillance. Decisions regarding modification or temporary interruption of BMA therapy should be individualised. Although the mandibular BSIJmax cut‐off value of 0.033 identified in this study may provide a useful reference, treatment decisions should carefully balance the benefits of preventing SREs against the risk of MRONJ in each patient. Rather than serving as a sole determinant for altering systemic therapy, BSIJmax should function as an adjunctive tool prompting multidisciplinary risk assessment—including collaboration with dental specialists—and facilitating earlier preventive measures. Integration of serial BSIJmax monitoring into existing preventive strategies, such as baseline dental assessment, avoidance of elective invasive dental procedures and patient education, may improve risk stratification without compromising oncologic outcomes. This approach aligns with current AAOMS guidelines, which advocate for risk‐based surveillance and proactive dental management in patients who are high‐risk.

This study had several limitations. First, this was a single‐centre, retrospective analysis with a relatively modest sample size, thereby necessitating validation in larger multicenter cohorts. Second, the number of patients with maxillary MRONJ was limited. Third, the inclusion of all MRONJ stages (0–3) in this study may have contributed to increased heterogeneity in the dataset. Finally, the imaging intervals were not standardised, which resulted in variability in the time between bone scintigraphy and diagnosis of MRONJ across patients.

In conclusion, BSIJmax is a promising, noninvasive and objective parameter for the early detection of MRONJ in patients with prostate cancer and bone metastases. Their incorporation into routine bone scintigraphy protocols may facilitate the timely identification of those who are high‐risk, enabling preventive measures that can improve clinical outcomes. Future prospective multicenter studies are warranted to validate the optimal cut‐off values and establish their roles in comprehensive MRONJ surveillance strategies.

## AUTHOR CONTRIBUTIONS

*Conceptualization:* Masaru Tani and Koji Hatano. *Methodology:* Masaru Tani, Koji Hatano, Takashi Kamiya, Tadashi Watabe and Kayako Isohashi. *Data curation:* Masaru Tani, Takashi Kamiya, Tadashi Watabe and Kayako Isohashi. *Formal analysis:* Masaru Tani. *Investigation:* Masaru Tani, Koji Hatano, Masatoshi Konishi, Atsuki Matsukawa, Hiromu Horitani, Syunsuke Inoguchi, Tomohiro Kanaki, Akihiro Yoshimura, Yuki Horibe, Yutong Liu, Nesrine Sassi, Toshiki Oka, Yohei Okuda, Gaku Yamamichi, Yu Ishizuya, Takuji Hayashi, Yoshiyuki Yamamoto, Taigo Kato, Tadashi Watabe, Atsunari Kawashima, Kayako Isohashi and Norio Nonomura. *Visualization:* Masaru Tani. *Writing—original draft:* Masaru Tani and Koji Hatano. *Writing—review and editing:* Masaru Tani, Koji Hatano, Takashi Kamiya, Tadashi Watabe and Kayako Isohashi. *Supervision:* Koji Hatano, Kayako Isohashi and Norio Nonomura. *Project administration:* Koji Hatano. *Funding acquisition:* Masaru Tani and Koji Hatano.

## CONFLICT OF INTEREST STATEMENT

The authors declare no competing interests.

## Supporting information


**Figure S1** Study schematic.

**Figure S2**Subgroup analysis of maxillary and mandibular bone scan index in the jaw (BSIJmax) according to type of bone‐modifying agents (BMAs) (A) Zoledronic acid (*n* = 79). (B) Denosumab (*n* = 56).

**Figure S3** Longitudinal changes in the maximum bone scan index in the jaw (BSIJmax). (A) The overall cohort (*n* = 37). (B) Patients treated with zoledronic acid (*n* = 28). (C) Patients treated with Denosumab (*n* = 9).

## Data Availability

The raw data were generated at Osaka University. Data supporting the results of this study are available upon request from the corresponding author.
