# Supplementary material for: Bone scan index rise prior to osteonecrosis of the jaw with bone‐modifying agents in prostate cancer
Source: BJUI Compass. 2026 Apr 28;7(5):e70212. doi: 10.1002/bco2.70212 (PMC13124443; doi:10.1002/bco2.70212)
Supplement: Supplementary file 1 — Figure S1 Study schematic. [file BCO2-7-e70212-s002.docx]

**Fig.S1** Study schematic
