# Supplementary material for: Bone scan index rise prior to osteonecrosis of the jaw with bone‐modifying agents in prostate cancer
Source: BJUI Compass. 2026 Apr 28;7(5):e70212. doi: 10.1002/bco2.70212 (PMC13124443; doi:10.1002/bco2.70212)
Supplement: Supplementary file 2 — Figure S2Subgroup analysis of maxillary and mandibular bone scan index in the jaw (BSIJmax) according to type of bone‐modifying agents (BMAs) (A) Zoledronic acid (n = 79). (B) Denosumab (n = 56). [file BCO2-7-e70212-s003.docx]

**Fig.S2** Subgroup analysis of maxillary and mandibular bone scan index in the jaw (BSIJmax) according to type of bone-modifying agents (BMAs)

(A) Zoledronic acid (n=79); (B) Denosumab (n=56)
