# Supplementary material for: Bone scan index rise prior to osteonecrosis of the jaw with bone‐modifying agents in prostate cancer
Source: BJUI Compass. 2026 Apr 28;7(5):e70212. doi: 10.1002/bco2.70212 (PMC13124443; doi:10.1002/bco2.70212)
Supplement: Supplementary file 3 — Figure S3 Longitudinal changes in the maximum bone scan index in the jaw (BSIJmax). (A) The overall cohort (n = 37). (B) Patients treated with zoledronic acid (n = 28). (C) Patients treated with Denosumab (n = 9). [file BCO2-7-e70212-s001.docx]

**Fig.S3** Longitudinal changes in the maximum bone scan index in the jaw (BSIJmax)

(A) The overall cohort (n=37) (B) Patients treated with zoledronic acid (n=28) (C) Patients treated with Denosumab (n=9) Significance values: * P < 0.05, ** P < 0.01, *** P < 0.001
